# Tobacco supply and demand strategies used in African countries

**DOI:** 10.2471/BLT.20.266932

**Published:** 2021-05-04

**Authors:** Eric Crosbie, Vincent Defrank, Catherine O Egbe, Olalekan Ayo-Yusuf, Stella Bialous

**Affiliations:** a School of Community Health Sciences, University of Nevada, 1664 N. Virginia Street, Reno, NV 89557, United States of America (USA).; b Alcohol, Tobacco and Other Drug Research Unit, South African Medical Research Council, Pretoria, South Africa.; c Africa Centre for Tobacco Industry Monitoring and Policy Research, Sefako Makgatho Health Sciences University, Pretoria, South Africa.; d School of Nursing, University of California, San Francisco, USA.

The World Health Organization (WHO) estimates that smoking kills more than 8 million people every year. While the WHO African Region has the lowest smoking prevalence of all WHO regions (roughly 6% of the world’s smokers), transnational tobacco companies are aggressively investing and marketing their products in the continent, hindering efforts towards lowering tobacco use prevalence. Currently, over 77 million adults smoke in Africa and this figure is expected to grow steadily over the next century, reaching 413 million smokers by 2100.^1^ A more immediate concern is that the number of smokers in Africa (based on 2010 levels) is anticipated to rise from 15.8% to 21.9% by 2030, the largest projected increase in the world.^2^ Here we focus on the main tobacco companies operating in Africa: Philip Morris International, British American Tobacco, Imperial Brands and Japan Tobacco International, and on their attempts to increase the supply and demand of tobacco to the African market.

## Illicit trade and smuggling

Each of the tobacco companies, but most notably Philip Morris and British American Tobacco, have a long history of participating in the illicit trade of tobacco to force market entry into new and emerging markets, including Asia, Eastern Europe and Latin America.^3^ Tobacco companies spread misinformation surrounding illicit tobacco trade and purposely overestimate the extent of illicit tobacco trade to dissuade governments from adopting tobacco control measures such as tobacco tax increases and pictorial health warning labels.^4^ However, previous research analysing internal tobacco industry documents from British American Tobacco reveal that the company has been involved in smuggling in at least 40 of the 54 African countries.^5^ These smuggling trends began in the 1980s and continue to persist.^6^ Recent media reports document an upsurge in smuggled cigarettes, most notably of popular brands such as Philip Morris’ Marlboro and Japan Tobacco International’s Camel cigarettes from the two leading market share tobacco companies in the region,^7^ in several countries including Benin, Cameroon, Central African Republic, Egypt, Eritrea, Ethiopia, Gabon, Morocco and South Africa.^2^^,^^4^^,^^6^ In Morocco, a recent study reports that one out of eight cigarettes is illicit and mostly smuggled from neighbouring Algeria.^6^ For example, a legal pack of Marlboro cigarettes is sold in Morocco for 3.38 United States dollars (US$) while the same smuggled packs from Algeria and Mauritania cost only US$ 1.91 and US$ 1.35 per pack, respectively.^6^

An important tactic that tobacco companies continue to use to prevent policy measures designed to stop the supply side of illicit tobacco trade is entering into voluntary partnerships with law enforcement and custom agencies in the form of signing memoranda of understanding. Such documents have been signed by all four of the tobacco companies in Botswana, Eswatini, Lesotho, Mozambique, Namibia and South Africa, as well as by British American Tobacco and Phillip Morris in Ghana and by British American Tobacco in Egypt, Ghana, Nigeria, Mauritius and Zambia.^8^ These memoranda of understanding have helped establish productive relations with governments and helped pre-empt more restrictive government regulations concerning illicit trade.^8^ Such agreements, because of their lack of transparency and enforcement mechanisms, also undermine the Protocol to Eliminate Illicit Trade in Tobacco Products, which entered into force in 2018 and requires the establishment of a global tracking and tracing system, supply chain licensing and special investigative techniques. As of April 2021, less than half of the African countries (20 out of 54) were parties to protocol, leaving several countries in the region without the proper commitment, collaboration and enforcement mechanisms to tackle illicit tobacco trade.

## Selling individual cigarettes 

A growing number of countries have passed laws prohibiting the sale of individual cigarette sticks and require that cigarettes be sold in packs of 20, but governments do not effectively enforce these laws, creating an opportunity for tobacco companies. Tobacco companies encourage informal traders who sell single individual sticks of cigarettes by providing them with branded kiosks and umbrellas and mobilizing them to lobby against regulations that would ban the sale of single stick cigarettes.^9^ Tobacco companies overlook the sale of individual cigarette sticks in African countries to make them more accessible and attract young and low-income customers as well as to avoid health warning labels presented on cigarette packages.^9^ In 2016, approximately 38% of cigarette consumption from Morocco came from individual cigarette purchases.^6^^,^^9^

In countries such as Benin, Ethiopia, Niger, Nigeria and Senegal, individual cigarettes are commonly sold, despite laws that prohibit this practice.^9^ Due to relaxed regulations and enforcement in Ethiopia, for example, shopkeepers sell individual cigarettes and if questioned, allege that they did not know they had to stop selling individual sticks to minors and thought that children were purchasing cigarettes for their parents.^10^ In Benin, Burkina Faso, Cameroon, Nigeria and Uganda, individual cigarette sticks are sold in push carts in grocery stores and convenience stores located in close proximity to schools.^10^ In one survey, all schools in Benin, Burkina Faso and Cameroon reported individual cigarettes sold in close proximity of the school; in Uganda this figure was estimated to be close to 95% and in Nigeria close to 70%.^10^ The rise in illicit trade is also in part due to the selling of individual cigarettes, as the reselling of individual sticks makes it difficult to track illicit cigarettes and trace the origin of cigarette purchases.^9^

## Promotional giveaways

Similar to other places around the world, tobacco companies are using promotional tactics, including price reductions, coupons and giveaways to increase the demand and usage of tobacco. In Botswana and Senegal, tobacco companies strategically place representatives to give away free cigarettes in malls, nightclubs and hotels, as well as free gifts including pens, ashtrays and other promotional material with logos of different tobacco brands printed on these items,^11^ despite laws prohibiting these practices. Consistent with worldwide trends, tobacco companies are aggressively targeting women, especially adolescent women, with free products that have images of success, sociability, beauty and feminine liberation, which has proven to increase the likelihood of adolescents becoming cigarette users.

Tobacco companies consistently claim on their websites, in the media and in policy circles that they aim to stop illicit tobacco trade and only market to adult smokers. However, similar to what they have done in other low- and middle-income countries,^3^ tobacco companies continue to circumvent legislation to prevent youth tobacco use through illicit tobacco trade, enabling individual cigarette sticks and providing promotional giveaways to recruit a new generation of smokers in Africa. Almost all of the countries in Africa have passed laws on preventing the sale of cigarettes to minors and several countries have put bans on tobacco promotions, but similar to Latin America and South Asia, enforcement remains problematic throughout the region. Thus, advocacy efforts should focus on enforcement and implementation strategies targeting the industry tactics discussed here. These efforts could include leveraging international funding from notable funders such as the Bloomberg Initiative to Reduce Tobacco Use and the Bill & Melinda Gates Foundation, and technical assistance from the WHO Regional Office for Africa towards these efforts; both have proven to be successful in other regions.^12^ Prioritizing enforcement of key supply-side and demand-side measures through the adoption and implementation of WHO’s Framework Convention on Tobacco Control – which guides comprehensive tobacco control policies – as well as the Protocol to Eliminate Illicit Trade in Tobacco Products, could help reduce this upsurge in new smokers throughout the African continent.

## References

[1] Blecher E, Ross H. Tobacco use in Africa: tobacco control through prevention. Atlanta: American Cancer Society; 2013. Available from: https://www.cancer.org/content/dam/cancer-org/cancer-control/en/reports/tobacco-use-in-africa-tobacco-control-through%3Dprevention.pdf [cited 2020 Jun 25].

[2] Boseley S. Threats, bullying, lawsuits: tobacco industry's dirty war for the African market. The Guardian. 2017 Jul 12. Available from: https://www.theguardian.com/world/2017/jul/12/big-tobacco-dirty-war-africa-market [cited 2019 Dec 10].

[3] Collin J, Legresley E, MacKenzie R, Lawrence S, Lee K. Complicity in contraband: British American Tobacco and cigarette smuggling in Asia. Tob Control. 2004 12;13 Suppl 2:ii104–11. 10.1136/tc.2004.009357 15564212 PMC1766170

[4] Blecher E. A mountain or a molehill: is the illicit trade in cigarettes undermining tobacco control policy in South Africa? Trends Organ Crime. 2010;13(4):299–315. 10.1007/s12117-010-9092-y

[5] Legresley E, Lee K, Muggli ME, Patel P, Collin J, Hurt RD. British American Tobacco and the “insidious impact of illicit trade” in cigarettes across Africa. Tob Control. 2008 10;17(5):339–46. 10.1136/tc.2008.025999 18617598 PMC2602753

[6] Masaiti A. 1 out of 8 Cigarettes in Morocco is illicit, mostly smuggled from Algeria. Morocco World News. 2017 Oct 23. Available from: https://www.moroccoworldnews.com/2017/10/231968/cigarettes-morocco-algeria-tobacco/ [cited 2019 Dec 20].

[7] Lambat I. Current tobacco industry dynamics in the Middle East and Africa. Tobacco Asia. 2020 Jul 6. Available from: https://www.tobaccoasia.com/features/current-tobacco-industry-dynamics-in-the-middle-east-and-afr/ [cited 2021 Jan 30].

[8] Crosbie E, Bialous S, Glantz SA. Memoranda of understanding: a tobacco industry strategy to undermine illicit tobacco trade policies. Tob Control. 2019 12;28 e2:e110–8. 10.1136/tobaccocontrol-2018-054668 30659106 PMC6639151

[9] Sale of single sticks of cigarettes in Africa: survey report from 10 capital cities. Lomé: Africa Tobacco Control Alliance; 2018. Available from: https://atca-africa.org/images/pdf/Atca-single-sticks/Report-Sale-of-Single-Sticks-in-Africa.pdf [cited 2020 Feb 2].

[10] Ashall F. Behind the smokescreen of Ethiopia’s surging tobacco production. Addis Standard. 2015 Oct 8. Available from: https://addisstandard.com/behind-the-smokescreen-of-ethiopias-surging-tobacco-production/ [cited 2020 Jan 10].

[11] Botswana citizens lead the pack in tobacco use - Official. African News. 2018 Jan 6. Available from: http://www.xinhuanet.com/english/2018-06/01/c_137222324.htm [cited 2019 Dec 20].

[12] Crosbie E, Sosa P, Glantz SA. Defending strong tobacco packaging and labelling regulations in Uruguay: transnational tobacco control network versus Philip Morris International. Tob Control. 2018 3;27(2):185–94. 10.1136/tobaccocontrol-2017-053690 28336521 PMC5610601

